# Cardiac Fibrosis and Cardiac Fibroblast Lineage-Tracing: Recent Advances

**DOI:** 10.3389/fphys.2020.00416

**Published:** 2020-05-06

**Authors:** Xing Fu, Qianglin Liu, Chaoyang Li, Yuxia Li, Leshan Wang

**Affiliations:** School of Animal Sciences, Louisiana State University Agricultural Center, Baton Rouge, LA, United States

**Keywords:** cardiac fibroblast, lineage-tracing, fibrosis, heart, cardiac disease

## Abstract

Cardiac fibrosis is a common pathological change associated with cardiac injuries and diseases. Even though the accumulation of collagens and other extracellular matrix (ECM) proteins may have some protective effects in certain situations, prolonged fibrosis usually negatively affects cardiac function and often leads to deleterious consequences. While the development of cardiac fibrosis involves several cell types, the major source of ECM proteins is cardiac fibroblast. The high plasticity of cardiac fibroblasts enables them to quickly change their behaviors in response to injury and transition between several differentiation states. However, the study of cardiac fibroblasts *in vivo* was very difficult due to the lack of specific research tools. The development of cardiac fibroblast lineage-tracing mouse lines has greatly promoted cardiac fibrosis research. In this article, we review the recent cardiac fibroblast lineage-tracing studies exploring the origin of cardiac fibroblasts and their complicated roles in cardiac fibrosis, and briefly discuss the translational potential of basic cardiac fibroblast researches.

## Introduction

Cardiac fibrosis is commonly associated with cardiovascular diseases (CVDs), the leading cause of death in western countries. In the United States, the direct health expenditures and lost productivity associated with CVDs in 2010 was estimated to be $315.4 billion (Go et al., 2014). Cardiac fibrosis is mainly present in two forms which are replacement fibrosis and interstitial fibrosis. Replacement fibrosis is characterized by the formation of collagenous scar tissue replacing the dead cardiomyocytes, which is often observed after myocardial infarction (MI) (Segura et al., 2014). Interstitial fibrosis, in contrast, usually does not involve massive cardiomyocyte death and is characterized by the accumulation of ECM proteins in the interstitial space between cardiomyocytes, which may be caused by pressure overload and infection (Fenoglio et al., 1983; Creemers and Pinto, 2010). In both forms of cardiac fibrosis, the activation of cardiac fibroblasts is involved (Ivey et al., 2018). The mechanical stress and inflammation stimulate cardiac fibroblast proliferation and promote their differentiation into myofibroblasts with elevated expression of ECM proteins (Ivey and Tallquist, 2016; Tallquist and Molkentin, 2017). Besides the accumulated ECM which stiffens the heart, the fibrotic tissue and activated fibroblasts also affect the contraction of adjacent cardiomyocytes, leading to the reduction of cardiac function (Kong et al., 2014). However, despite the deleterious effects of fibrosis on cardiac function, the accumulated ECM and myofibroblasts filled with stress fibers may play an important role in maintaining tissue integrity especially when a substantial death of cardiomyocytes occurs (Talman and Ruskoaho, 2016). In addition, cardiac fibroblasts may also regulate cardiac function through cross-talking with other cell types residing in the heart such as cardiomyocytes (Zhang et al., 2012) and immune cells (Van Linthout et al., 2014). Due to the complicated role of cardiac fibroblasts in the diseased heart, manipulation of cardiac fibroblast activities to promote their beneficial effects and limit their deleterious effects is needed, which requires a complete understanding of the regulation of cardiac fibroblast activities. The development of cardiac fibroblast lineage-tracing mouse lines promoted researches addressing many fundamental questions about cardiac fibroblast. This review summarizes the recent advances in cardiac fibroblast lineage-tracing and the findings resulted from researches utilizing these tools. The clinical implication of cardiac fibroblast lineage-tracing studies is also briefly discussed.

## Cardiac Fibroblast Markers

By definition, fibroblasts are a group of cells secreting collagen and other ECM proteins. They reside in the interstitial spaces between tissue parenchymal cells. Under basal conditions, these cells stay mostly quiescent and likely play important roles in maintaining the interstitial connective tissue and mediating the communication between surrounding cells (Ivey and Tallquist, 2016; Tallquist and Molkentin, 2017). Under stressed conditions, their production of ECM proteins significantly increases, which is responsible for the formation of fibrotic tissue (Souders et al., 2009; Ivey and Tallquist, 2016; Tallquist and Molkentin, 2017). Thus, many identified fibroblast markers are ECM proteins. Some ECM proteins highly expressed by fibroblasts are fibrillar collagens such as collagen I and collagen III and fibronectin (Carver et al., 1991; Philips et al., 1994). The expression of these ECM proteins significantly increases after the differentiation of cardiac fibroblasts into myofibroblasts (Baum and Duffy, 2011). In addition, myofibroblasts express some unique ECM proteins such as periostin (Conway and Molkentin, 2008; Shimazaki et al., 2008), and ECM remodeling enzymes such as matrix metalloproteases (MMPs), tissue inhibitors of metalloproteinases (TIMPs), and lysyl oxidase (LOX) (Polyakova et al., 2004; Brown et al., 2007; Fan et al., 2012; Lindner et al., 2012; El Hajj et al., 2017). Similar to other types of cells, a cytoskeletal network is present in fibroblasts. While many components in this network are common proteins also expressed in other cell types, some of them have been used as fibroblast markers due to their unique expression in fibroblasts. Vimentin, an intermediate filament protein, is abundant in both quiescent fibroblasts and myofibroblasts (Wang et al., 2003; Baum and Duffy, 2011). In contrast, smooth muscle α-actin (αSMA, encoded by *Acta2*) is exclusively expressed after myofibroblast differentiation (Leslie et al., 1991). Another group of fibroblast markers are transcription factors that are specifically expressed in fibroblasts. Transcription factors that have been used as cardiac fibroblast markers include Tcf21, WT1, Tbx18, and Tie2 (Quaggin et al., 1999; Cai et al., 2008; Zeisberg and Kalluri, 2010; Zhou and Pu, 2012; Moore-Morris et al., 2014). Developmentally, epicardial fibroblast progenitors express Tcf21, WT1, and Tbx18, while Tie2 expression has only been identified in endocardial cardiac fibroblast progenitors. Studies have also reported cell surface markers of fibroblasts, which are important due to their potential use in cell sorting (Smith et al., 2011; Asli et al., 2019). Among these identified cell surface markers, platelet-derived growth factor receptor alpha (PDGFRα) is the most widely used that is abundantly expressed in fibroblasts found in various tissues (Smith et al., 2011; Asli et al., 2019). Other cell surface markers that have been reported to be expressed by fibroblasts include NG2 and PDGFRβ which, however, are primarily expressed in pericytes which are fibroblast-like cells associated with blood vessels (Chen et al., 2016).

## Cardiac Fibroblast Lineage-Tracing Systems and Tools

The definition of lineage-tracing is the identification of all progeny of a single cell or cell population. The principle of lineage-tracing is that the expression of reporter genes under the control of the *cis*-regulatory elements of a gene that is specifically expressed in a cell type permits the identification of this cell population and its descendants. Thus, the identification of fibroblast markers has facilitated the development of some fibroblast lineage-tracing tools. However, not all previously identified fibroblast markers are suitable for fibroblast lineage-tracing because most of those markers are not exclusive to cardiac fibroblasts. The currently widely used lineage-tracing systems can be classified into two categories. The first category utilizes a reporter gene, such as GFP, directly controlled by a tissue-specific promoter or locus, which is referred to as direct lineage-tracing in this article. The reporter gene can be introduced into the genome as a transgene together with a tissue-specific promoter or inserted into a tissue-specific locus (knock-in) (Hamilton et al., 2003; LeBleu et al., 2013). The other category employs the Cre-loxP recombination system (Sauer, 1998) which includes two components, a Cre recombinase (Cre) gene driven by a tissue-specific promoter/locus and a reporter gene downstream of a stop codon flanked by two loxP sites (Soriano, 1999). The expression of Cre induces the deletion of the stop codon between the two loxP sites, leading to the expression of the reporter gene, which is thus referred to as indirect lineage-tracing. Similarly, Cre can be introduced into the genome together with a tissue-specific promoter as a transgene or knocked into a specific tissue-specific locus. In the Cre-loxP system, the reporter gene is usually inserted into the ROSA26 locus and under the control of a strong promoter (Madisen et al., 2010). Multiple Cre isoforms have been developed by different researchers. While the original Cre becomes active once being expressed (Soriano, 1999), the Cre mutants fused to one (CreERT and CreERT2) or two (MerCreMer) mutated murine estrogen-binding domains are only active when bound to 4-hydroxy-tamoxifen (Metzger et al., 1995; Zhang et al., 1996; Indra et al., 1999). CreERT2 is the second generation of tamoxifen-inducible Cre developed based on CreERT and is more sensitive to 4-hydroxy-tamoxifen as compared to CreERT (Indra et al., 1999). The MerCreMer mediates more stringent recombination likely because each MerCreMer requires two, rather than one, 4-hydroxy-tamoxifen molecules for activation (Verrou et al., 1999).

The major advantage of the direct control of the report gene expression by a tissue-specific promoter/locus is that the expression level of the reporter usually reflects the promoter activity, which enables the tracking of the expression of the tissue-specific gene. However, when the activity of the tissue-specific promoter is low, the expression of the reporter may not be strong enough for certain experiments. In addition, the activity of some promoters or loci may totally disappear or start in a particular developmental or disease stage. Thus, the reporter-labeled cells identified at a time point may not be the same population of cells observed at an earlier time point or their descendants. Examples of this system include *αSMA-RFP* mice (Magness et al., 2004), *Col1a1-GFP* mice (Kalajzic et al., 2002; Yata et al., 2003), *Tcf21-lacZ* (Quaggin et al., 1999), *Pdgfra-GFP* (Hamilton et al., 2003), and *Postn-lacZ* (Snider et al., 2008). In contrast to the direct lineage-tracing system, one common advantage of the indirect lineage-tracing system is that the expression of the reporter in a cell and all its descendants is permanent once the recombination has taken place regardless of the change in the activity of the promoter driving the Cre, which is favored in some studies. Lineage-tracing mouse lines of this category that have been used in cardiac fibroblast studies include *Acta2-CreERT2* (Wendling et al., 2009), *Col1a2-CreERT* (Ubil et al., 2014), *Col1a1-CreERT2* (Biswas, 2016), *Postn-CreERT2* (Kaur et al., 2016), *Tbx18-Cre* (Cai et al., 2008), *Tbx18-CreERT2* (Moore-Morris et al., 2014), *Tcf21-Cre* (Acharya et al., 2011), *Tcf21-MerCreMer* (Acharya et al., 2011), *Tie2-Cre* (Kisanuki et al., 2001), *Sox9-CreER* (He et al., 2017), *WT1-Cre* (Moore-Morris et al., 2014), and *WT1-CreERT2* (Ali et al., 2014; Moore-Morris et al., 2014). The choice between a non-inducible Cre and an inducible Cre depends on the goal of the study. A unique feature of the lineage-tracing system using non-inducible Cre is that the reporter expression starts once the promoter driving the Cre becomes active. This can be an advantage in some developmental studies focusing on the fate of cells derived from an embryonic lineage. However, such a feature can be a problem in some studies aimed at lineage-tracing cells expressing a certain gene at a particular time point as some of the lineage-traced cells observed in non-inducible Cre lines may be a result of a previous recombination and may no longer express the gene whose promoter drives the Cre, which may lead to a false conclusion. The inducible Cre, on the other hand, allows the timely control of recombination. A drawback of the inducible Cre is the lower recombination efficiency as compared with non-inducible Cre. Repeated or continuous tamoxifen treatment is required to induce sufficient recombination. However, some side effects associated with tamoxifen have been reported and may affect the experimental result. In particular, tamoxifen often induces dystocia when applied to pregnant female mice (Narver, 2012). The retrieval of the pups requires cesarean sections which are very labor-intense. Moreover, it has been shown that tamoxifen can induce physiological changes such as the browning of adipose tissue in female mice (Zhao et al., 2019), likely due to its anti-estrogenic activity, which may affect the experimental results.

As mentioned previously in this article, both the reporter gene in the direct lineage-tracing system and the Cre in the indirect lineage-tracing system can be introduced into the animal together with the promoter as a transgene randomly inserted into the genome or specifically knocked into a locus that is only active in certain cell type. Some examples of the mouse lines generated using the transgene strategy are *αSMA-RFP* (Magness et al., 2004), *Col1a1-GFP* (Kalajzic et al., 2002; Yata et al., 2003), *Col1a2-CreERT* (Ubil et al., 2014), *Col1a1-CreERT2* (Biswas, 2016), *Tie2-Cre* (Kisanuki et al., 2001), and *αSMA-CreERT2* (Wendling et al., 2009). The major advantage of the transgene strategy is that it is relatively simple to generate a lineage-tracing mouse line in this way. However, the efficiency and reliability of these lines vary significantly. The reason is that the included promoter may lack certain regulatory elements. For example, among the 3 *Col1a1-GFP* mouse lines, only the one containing the collagen gene promoter (−3122 to +111) and upstream DNase I-hypersensitive sites has been shown to specifically and efficiently label cardiac fibroblasts (Yata et al., 2003), while GFP expression was absent in the heart of the other *Col1a1-GFP* mouse lines (Kalajzic et al., 2002). Moreover, *αSMA-CreERT2* efficiently induced reporter expression in cardiac myofibroblast after MI (Fu et al., 2018), however, the *αSMA-RFP* mouse line failed to do so (personal experience). In addition, the random insertion of the transgene may disrupt endogenous gene expression causing unintended mutation. In contrast, the knock-in strategy usually allows the precise control of the reporter or Cre expression by the endogenous tissue-specific locus, which is desired in most lineage-tracing studies. Examples of cardiac fibroblast lineage-tracing mouse lines created using this strategy include *Postn-CreERT2* (Kaur et al., 2016), *Tbx18-Cre* (Cai et al., 2008), *Tbx18-CreERT2* (Moore-Morris et al., 2014), *Tcf21-Cre* (Acharya et al., 2011), *Tcf21-MerCreMer* (Acharya et al., 2011), *Postn-MerCreMer* (Kanisicak et al., 2016), *WT1-Cre* (Moore-Morris et al., 2014), and *WT1-CreERT2* (Ali et al., 2014; Moore-Morris et al., 2014). However, the knock-in of the Cre also abolishes the expression of the endogenous gene expression from the disrupted allele. Even though homozygous knockout can be avoided through genotyping, the heterozygous knockout may also have a milder effect.

Recently, the Dre-rox, a system similar to Cre-loxP (Anastassiadis et al., 2009), has been applied in lineage-tracing studies of cardiac fibroblasts and other cell types (He et al., 2017; Pu et al., 2018). The expression of Dre induces the recombination of rox sites, resulting in the expression or knockout of a gene. In addition to the original Dre allele, Dre fused to murine estrogen-binding domains has also being generated, which enables tamoxifen-dependent Dre activity (He et al., 2017). More important, due to the high specificity of Cre-loxP and Dre-rox systems, the dual lineage-tracing combining these two systems is a recent trend in lineage-tracing studies. Mice carrying both Cre-loxP and Dre-rox permit the simultaneous tracing of two distinct or related cell types (Liu et al., 2019). These mice may be particularly useful in the study of cardiac fibroblast differentiation and transdifferentiation when the precursor cells and their descendants express different marker genes.

Besides the use of cardiac fibroblast lineage-tracing mouse lines to understand the origin of cardiac fibroblasts and their cell fate changes in response to cardiac injuries, an equally, if not more, important application of these tools is the study of mechanisms regulating cardiac fibroblast activities after injuries and their therapeutic potentials. When cross-bred with a mouse line carrying a gene with its essential exon(s) flanked by *loxP* sites or a Cre-dependent gene overexpression cassette containing a 5′ *loxP*-flanked stop sequence, cardiac fibroblast-specific knockout or overexpression of the gene can be achieved *in vivo*. Importantly, even though many of the aforementioned Cre lines have been utilized to study the cardiac fibroblast-specific functions of genes, they do have different advantages and limitations largely due to the variation in the activities of promoters driving Cre expression. For example. *Tcf21-MerCreMer* mouse line has been successfully employed to lineage-trace cardiac fibroblasts during development and in normal hearts and study cardiac fibroblast differentiation after cardiac injuries (Acharya et al., 2011; Kanisicak et al., 2016; Khalil et al., 2017; Ivey et al., 2018). However, the ability of this line to specifically induce gene knockout or overexpression in myofibroblasts may be limited as the expression of *Tcf21* in cardiac fibroblasts dramatically decreases during myofibroblast differentiation (Fu et al., 2018). As a result, pre-injury tamoxifen treatment to mouse lines carrying *Tcf21-MerCreMer* is usually required for successful and efficient recombination (Khalil et al., 2017; Xiang et al., 2017). However, the myofibroblast-specific expression of the target genes may render pre-injury tamoxifen treatment less efficient in these lines, as the condensed chromatin may reduce the accessibility of Cre to *loxP* sites in the genes that are not being actively expressed. In contrast, mouse lines such as *Postn-MerCreMer* and *Col1a2-CreERT2* are likely more efficient in inducing gene knockout and overexpression in myofibroblasts due to their high level of expression in these cells (Fu et al., 2018). And combining two Cre lines, such as *Tcf21-MerCreMer* and *Postn-MerCreMer* may grant more efficient and complete recombination in cardiac fibroblasts of different states.

## The Developmental Origin of Cardiac Fibroblasts

One of the most important fundamental questions about cardiac fibroblasts is their developmental origin. Studies utilizing *Tcf21* (*Tcf21-MerCreMer*), *Wt1* (*Wt1-Cre*), and *Tbx18* (*Tbx18-Cre*) *cis*-regulatory elements-controlled lineage-tracing mouse lines have shown that epicardial progenitors give rise to both cardiac fibroblasts and vascular smooth muscle cells developmentally (Cai et al., 2008; Zhou et al., 2008; Smith et al., 2011; Acharya et al., 2012; Moore-Morris et al., 2014), which is consistent with earlier experiments conducted using chick embryos (Mikawa and Gourdie, 1996). In mice, these epicardial cardiac fibroblast progenitors express PDGFRα and start the epithelial-to-mesenchymal transition (EMT) around embryonic day 14.5 (E14.5). Disruption of PDGFRα, Wt1, or Tcf21 signaling caused significantly defects in this process (Moore et al., 1999; Smith et al., 2011; Acharya et al., 2012). The requirement of Tbx18 in this process, however, has not been revealed. Using the *Tie2-Cre* lineage-tracing mouse line, it was shown that some cardiac fibroblasts were derived from endocardium through the endothelial-to-mesenchymal transition (EndoMT) (Kisanuki et al., 2001; Ali et al., 2014). In mouse heart, the EndoMT process of *Tie2* lineage-traced endocardial cells first starts around E11.5 day in the outflow septum (Kisanuki et al., 2001). It was estimated that around 85% of cardiac fibroblasts are derived from epicardial cells, while the other ∼15% are derived from endothelial cells (Moore-Morris et al., 2014). Moreover, a difference in the spatial distribution of these two cardiac fibroblast subpopulations has been noticed. While endocardium-derived cardiac fibroblasts are more prevalent in the interventricular septum, the ones derived from epicardium are more dominant in the left ventricular free wall (Moore-Morris et al., 2014). It is worth noting that the *Wt1-Cre* mice and *Tie2-Cre* mice used in this study also labeled cardiomyocytes and endothelial cells, respectively. Moreover, experiments treating adult *Tcf21-MerCreMer*, *WT1-CreERT2*, and *Tbx18-CreERT2* mice with tamoxifen showed that only *Tcf21* was still expressed in adult cardiac fibroblast while the lineage-traced cells in *WT1-CreERT2* and *Tbx18-CreERT2* mice were primarily epicardial cells (Moore-Morris et al., 2014), indicating the limitations of some of these lines.

## Cardiac Fibroblast Origin in the Diseased Heart

Many heart diseases trigger cardiac fibrosis. Both interstitial fibrosis and replacement fibrosis involves the activation of cardiac fibroblasts and their differentiation into myofibroblasts. As the major fibroblast lineages in the normal heart, it is not a surprise that endocardium and epicardium-derived resident cardiac fibroblasts are also the major contributors to the cardiac myofibroblast population in the injured heart (Ali et al., 2014; Moore-Morris et al., 2014; Kanisicak et al., 2016; Fu et al., 2018). Dramatic changes in cellular composition happen in the diseased heart. A question asked by many researchers is whether epicardial and endocardial cells in the adult heart still have the ability to transdifferentiate into cardiac fibroblasts in response to injury. While earlier studies suggested the existence of EMT in the adult heart (van Tuyn et al., 2007; Russell et al., 2011; Zhou and Pu, 2011; Duan et al., 2012), careful lineage-tracing studies using *WT1-CreERT2* and *Tbx18-CreERT2* mouse lines found that the adult epicardial cells do not give rise to cardiac fibroblasts after cardiac injury (Ali et al., 2014; Moore-Morris et al., 2014). In contrast, the study of the EndoMT of *Tie2* lineage-traced cells in the injured adult heart is still missing. This is largely due to the limitation of the *Tie2-Cre* mouse line (Kisanuki et al., 2001; Ali et al., 2014). Interestingly, in contrast to *Tcf21* lineage-traced cardiac fibroblasts which all differentiated into myofibroblasts expressing *Postn* in response to injury (Kanisicak et al., 2016; Fu et al., 2018), a study using another endothelial/endocardial lineage-tracing mouse line, *Cdh5-Cre*, found that less than 1% of *Cdh5* lineage-traced cells (including both endothelial cells and endothelium-derived cardiac fibroblasts) expressed *Postn* after MI (Kanisicak et al., 2016), suggesting a difference in the transcriptomic regulation of cardiac fibroblasts of different origins. It is possible that the EndoMT taken place in some of the endothelial/endocardial cells during development is actually a partial EndoMT, causing an incomplete cell fate change (Li et al., 2018). However, a definitive answer about EndMT in the adult heart requires inducible endothelial lineage-tracing mouse lines such as *Tie2-CreERT2* and *Cdh5-CreERT* mice, both of which are available now (Wang et al., 2010; Maliken et al., 2018). The hematopoietic cell is another cell type that has been reported to transdifferentiate into cardiac fibroblasts in response to ischemic injury. Bone marrow transplantation experiments showed that bone marrow-derived cells positive for collagen I and αSMA were present in the infarct (Haudek et al., 2006; Möllmann et al., 2006; van Amerongen et al., 2008; Verma et al., 2017). However, studies using hematopoietic cell lineage-tracing mouse lines found that these cells minimally contributed to the cardiac fibroblast population (Ali et al., 2014; Moore-Morris et al., 2014; Kanisicak et al., 2016), suggesting that this transdifferentiation is a rather rare event. It is possible that the bone marrow-derived fibroblasts identified in the recipient mice in bone marrow transplantation experiments were actually derived from transplanted bone marrow mesenchymal progenitor cells instead of hematopoietic cells as myofibroblast differentiation of bone marrow mesenchymal progenitor cells has been reported (Quante et al., 2011). Thus, further lineage-tracing studies of bone marrow mesenchymal progenitor cells is required to answer this question.

## Cardiac Fibroblast Plasticity

In general, the fibroblast is a very plastic cell type. The differentiation of cardiac fibroblasts into myofibroblast has been verified by many groups using multiple cardiac fibroblast lineage-tracing lines (Ali et al., 2014; Moore-Morris et al., 2014; Kanisicak et al., 2016; Khalil et al., 2017; Fu et al., 2018). The transcriptional regulation of myofibroblast differentiation is very complicated as many signaling pathways including TGFβ and novel mechanisms have been revealed (see section “Targeting Cardiac Fibroblasts to Treat Heart Diseases” for more detailed discussion). It was once believed that myofibroblasts were terminal-differentiated and were cleared by apoptosis once the infarct scar was stabilized, which was evidenced by the disappearance of αSMA^+^ myofibroblasts (Takemura et al., 1998). However, our recent lineage-tracing study showed that *Tcf21* lineage-traced cardiac fibroblasts persisted in infarct scar beyond 8 weeks after MI (Fu et al., 2018). Myofibroblasts in stabilized infarct scar further differentiated into matrifibrocytes which did not express αSMA and only expressed low levels of typical myocardial ECM proteins such as collagen 1 and collagen 3 (Fu et al., 2018). Instead, matrifibrocytes expressed a high level of cartilage ECM proteins, suggesting a partial transcriptomic switch to chondrocyte (Fu et al., 2018). Removal of matrifibrocytes from infarct scar by cryoinjury caused a reduction in cardiac function and exacerbation of dilation, suggesting an important function of matrifibrocytes in maintaining the infarct stability (Fu et al., 2018). However, mechanisms responsible for matrifibrocyte differentiation are still not clear. Another study found that in mouse lines susceptible to injury-induced myocardial calcification, *Col1a2* lineage-traced cardiac fibroblasts differentiated into a calcium-depositing cell type similar to osteoblast, a process believed to be mediated by Runx2, an osteogenic transcription factor (Pillai et al., 2017). Moreover, it was recently shown that *Tcf21* lineage-traced cardiac fibroblasts differentiated into adipocytes in response to a high-fat diet (HFD), which is normally inhibited by cardiomyocyte-secreted prokineticin-2 (Qureshi et al., 2017), further suggesting the plasticity of these cells. The transdifferentiation of cardiac fibroblasts into endothelial cells upon MI has also been investigated. It was reported that ∼35% of *Col1a2* lineage-traced cardiac fibroblasts expressed endothelial markers such as VECAD and contributed to the neovascularization in injured hearts (Ubil et al., 2014), suggesting a novel therapeutic role of cardiac fibroblasts. However, the study conducted by another group using multiple cardiac fibroblast lineage-tracing lines including the same *Col1a2-CreERT* mouse line showed that the neovascularization process was mainly mediated by the expansion of pre-existing endothelial cells but not cardiac fibroblasts (He et al., 2017). The contradictory results of the two studies may be partially due to the difference in the markers and antibodies used for endothelial cell identification. A study using a dual lineage-tracing system may help clarify this contradiction.

## Targeting Cardiac Fibroblasts to Treat Heart Diseases

An ultimate goal of cardiac fibroblast lineage-tracing is to treat heart diseases through manipulating cardiac fibroblast activities. This requires the understanding of the functions of genes expressed in cardiac fibroblasts and their roles in disease development. These studies have been greatly promoted by Cre-loxP-mediated cardiac fibroblast lineage-tracing mouse lines, which allows specific knockout and overexpression of genes in cardiac fibroblasts *in vivo* without affecting other tissues/cell types (see Table 1 for a summary of recent relevant studies).

**TABLE 1:** Summarization of recent findings by employing Cre-loxP-mediated cardiac fibroblast-specific gene deletion or overexpression mouse lines.

**Cre lines used**	**Genes deleted or overexpressed**	**Injury model**	**Changes in cardiac fibroblasts**	**Impact on cardiac function and phenotype**	**References**
*Tcf21-MerCreMer; Postn-MerCreMer*	*Tgfbr1/2* double deletion; *Smad2/3* single and double deletion	TAC	Reduced myofibroblast proliferation, differentiation, contraction, and ECM protein expression	Improved function (Tgfbr1/2 deletion only); reduced fibrosis (all except Smad2 deletion)	Khalil et al., 2017
*Tcf21-MerCreMer*; *Postn-MerCreMer*	*Ctnnb1* deletion	TAC	Increased ECM gene expression	Improved function; reduced fibrosis	Xiang et al., 2017
*Tcf21-MerCreMer*	*Pkr1* deletion	HFD	Increased adipogenesis	Reduced function	Qureshi et al., 2017
*Tcf21-MerCreMer*	*Fn1* deletion	I/R	Reduced myofibroblast number	Improved function; reduced fibrosis and cardiomyocyte hypertrophy	Valiente-Alandi et al., 2018
*Tcf21-MerCreMer*	*Loxl2* deletion	TAC	Reduced TGF-β2 production and myofibroblast differentiation	Improved function; reduced fibrosis	Yang et al., 2016
*Tcf21-MerCreMer*	*Lats1/2* double deletion	None	Spontaneous myofibroblast differentiation	Reduced function; increased fibrosis	Xiao et al., 2019
*Postn-MerCreMer*	*Tgfbr2* deletion	Mouse model of proteotoxic cardiac disease	Reduced myofibroblast number	Improved function and survival rate; reduced cardiomyocyte hypertrophy	Bhandary et al., 2018
*Postn-MerCreMer*	*Tgfbr2* deletion	Mouse model of cardiac myosin binding protein c-induced cardiomyopathy	Reduced myofibroblast number	Improved function and survival rate; reduced fibrosis	Meng et al., 2018
*Postn-MerCreMer*	*Mapk14* deletion	MI; I/R	Reduced myofibroblast differentiation and expansion	Improved function; reduced fibrosis	Molkentin et al., 2017
*Postn-MerCreMer*	*Ccn2* deletion	Ang II infusion	Attenuated TGFβ-induced myofibroblast differentiation	Reduced fibrosis	Dorn et al., 2018
*Postn-MerCreMer*	*Hsp47* deletion	TAC; MI	Reduced collagen production	Improved function (TAC); reduced fibrosis (TAC and MI); increased rupture (MI); reduced function (MI)	Khalil et al., 2019
*Postn-MerCreMer*	*Grk2* deletion	I/R	Reduced myofibroblast differentiation	Improved function; reduced fibrosis	Travers et al., 2017
*Postn-Cre*	*Smad2* and *Smad3* deletion	MI; I/R	Enhanced proliferation; disorganized stress fibers (Smad3 deletion)	Temporary improved of function (Smad2 deletion); exacerbated long-term function and enchanced ECM maladaptive remodeling (Smad3 deletion)	Kong et al., 2018; Huang et al., 2019
*Postn-Cre*	*Rock2* deletion; *Rock2* overexpression	Ang II infusion	Reduced myofibroblast phenotype (Rock 2 deletion vs. overexpression)	Improved function and reduced fibrosis (Rock 2 deletion vs. overexpression)	Shimizu et al., 2017
*Postn-Cre*	*Pkd1* deletion	I/R; MI	Attenuated TGFβ-induced myofibroblast differentiation	Reduced scar size; increased cardiomyocyte hypertrophy	Villalobos et al., 2019
*Postn-Cre*	*Il17ra* deletion	MI	Reduced granulocyte macrophage colony-stimulating factor^+^ (GM-CSF^+^) fibroblast	Increased survival rate; reduced infarct size	Chen et al., 2018
*Postn-Cre*	*Klf5* deletion	TAC	Reduced IGF-1 secretion	Improved function; reduced cardiomyocyte hypertrophy	Takeda et al., 2010
*Postn-Cre*	*Sox9* deletion	MI	Reduced myofibroblast phenotype	Improved function; reduced dilation and scar size	Scharf et al., 2019
*Postn-Cre*	*miR-33* deletion	TAC	Reduced proliferation and lipid raft content	Reduced fibrosis	Nishiga et al., 2017; Dorn et al., 2018
*Col1a2-CreERT*	*Atf3* overexpression	Ang II infusion	Reduced p38 activation	Improved function; reduced fibrosis	Li et al., 2017
*Col1a2-CreERT*	*Il1r1* deletion	MI	Reduced IL-1α–induced Il6, Mmp3, and Mmp9 expression	Improved function; reduced fibrosis	Bageghni et al., 2019
*Col1a2-CreERT*	*Il11* overexpression	None	Increased myofibroblast differentiation	Reduced function; increased fibrosis	Schafer et al., 2017
*Col1a2-CreERT*	*Grk2* deletion	I/R	Reduced TNFα secretion	Improved function; reduced fibrosis and inflammation	Woodall et al., 2016
*Col1a2-CreERT*	*Mcu* deletion	MI; Ang II infusion	Increased myofibroblast differentiation	Reduced function; increased fibrosis	Lombardi et al., 2019

*Studies were grouped based on Cre mouse lines used.*

### Targeting TGFβ and Related Signaling

The correlation between TGFβ signaling and cardiac fibrosis has long been noticed (Casscells et al., 1990). Only recently, with the help of cardiac fibroblast lineage-tracing and specific gene knockout, many details in this process have been revealed *in vivo*. Using *Tcf21-MerCreMer* and *Postn-MerCreMer*-mediated cardiac fibroblast-specific knockout of *Tgfbr1/2 Smad2* and *Smad3*, which are important components of canonical TGFβ signaling, we found that in cardiac fibroblast canonical TGFβ signaling played an important role in activating cardiac fibroblasts, which was mainly mediated by Smad3 but not Smad2 (Khalil et al., 2017). Interestingly, even though both cardiac fibroblast-specific deletion of *Tgfbr1/2* and *Smad2/3* significantly reduced cardiac fibrosis induced by transverse aortic constriction (TAC), only the deletion of *Tgfbr1/2* in cardiac fibroblasts ameliorated cardiac hypertrophy and resulted in a functional improvement at 4 and 12 weeks after TAC (Khalil et al., 2017). Similarly, myofibroblast-specific *Tgfbr2* deletion led to improved cardiac function in mouse models of proteotoxic cardiac disease and cardiac myosin binding protein C-induced cardiomyopathy (Bhandary et al., 2018; Meng et al., 2018). Studies conducted by another group using *Postn-Cre*-mediated myofibroblast-specific deletion of *Smad3* and *Smad2* found that while deletion of *Smad2* temporarily improved cardiac function around 7 days after myocardial infarct (MI) in mice, deletion of *Smad3* caused a reduction in cardiac function 28 days after MI (Kong et al., 2018; Huang et al., 2019). The identified difference between *Smad2* and *Smad3* deletion was likely because of the differential regulation of myofibroblast and collagen matrix organization by Smad2 and Smad3 (Kong et al., 2018; Huang et al., 2019; Russo et al., 2019). The limited controversies in the effect of Smad gene deletion on cardiac function reported in these studies were possibly due to the different injury models used in which the expression of Smad2/3 in cardiac fibroblasts may have different effects on cardiac function. Moreover, the phenotypic and functional differences between mice with cardiac fibroblast-specific deletion of *Tgfbr1/2* and *Smad2/3* suggest that non-canonical TGFβ signaling also plays an important role in cardiac fibroblast-mediated post-injury remodeling (Khalil et al., 2017). Indeed, using the same cardiac fibroblast-specific Cre lines, the role of TGFβ-MAPK kinase 6 (MKK6)-p38 signaling in myofibroblast differentiation of cardiac fibroblasts was revealed (Molkentin et al., 2017). Cardiac fibroblast-specific deletion of *Mapk14* (the gene encoding p38) significantly reduced myofibroblast expansion and cardiac fibrosis and improved cardiac function after ischemia/reperfusion (I/R) in mice (Molkentin et al., 2017). A similar protective effect was observed in mice with cardiac fibroblast-specific deletion of *Atf3*, a transcription factor that activates the expression of *Map2k3*, a p38 kinase (Li et al., 2017). Besides, the TGFβ-RhoA-Rock pathway has also been found to play an important role in the actin stress fiber formation, which is essential for myofibroblast differentiation (Bhowmick et al., 2001; Shi and Massagué, 2003; Costanza et al., 2017). A study found that in angiotensin II (Ang II) infusion-induced cardiomyopathy, the myofibroblast-specific deletion of *Rock2* significantly reduced αSMA expression in these cells, together with reduced connective tissue growth factor (CTGF) and FGF2 expression in cardiac fibroblasts, and improved cardiac function and fibrosis (Shimizu et al., 2017). However, how the disrupted stress fiber formation led to reduced cytokine production is still not clear.

Besides the direct regulatory roles of canonical and non-canonical TGFβ signaling in the myofibroblast differentiation of cardiac fibroblasts and cardiac fibrosis, the cross-talk between TGFβ signaling and other signaling pathways has also been studied using fibroblast-specific Cre mouse lines. CTGF is an important mediator in TGFβ-induced myofibroblast differentiation (Mori et al., 1999; Shi-wen et al., 2006). Interestingly, despite the fact that cardiomyocyte is the major source of CTGF in the heart, the deletion of *Ccn2* (the gene encoding CTGF) in cardiomyocytes failed to protect the heart from pressure overload-induced cardiomyopathy (Accornero et al., 2015). A later study found that CTGF functioned as an autocrine factor in cardiac fibroblasts and cardiac fibroblast-specific deletion of *Ccn2* significantly improved cardiac function after Ang II infusion (Dorn et al., 2018). It was demonstrated that Wnt signaling and TGFβ signaling also cross-talk during myofibroblast differentiation (Chen et al., 2011; Akhmetshina et al., 2012). The elevated level of TGFβ in injured myocardium activated Wnt signaling which led to cardiac fibrosis in experimental autoimmune myocarditis (Blyszczuk et al., 2016). In a recent study specifically deleting *Ctnnb1* (the gene encoding β-catenin) in cardiac fibroblasts, the cardiac function and fibrosis were found to be improved, which was primarily due to reduced ECM protein secretion by cardiac fibroblasts (Xiang et al., 2017). Moreover, it was recently found that canonical TGFβ signaling during myofibroblast differentiation of cardiac fibroblasts required functional primary cilia in cardiac fibroblasts. When primary cilium is disrupted in cardiac fibroblasts due to cardiac fibroblast-specific *Pkd1* deletion, the TGFβ-Smad3 signaling-induced ECM protein production and myofibroblast differentiation of cardiac fibroblasts were impaired, leading to insufficient scar formation and increased cardiomyocyte hypertrophy after MI (Villalobos et al., 2019). Interleukin (IL) is an important group of inflammatory cytokines. Multiple IL receptors are expressed in cardiac fibroblasts, which regulates myofibroblast activities (Shinde and Frangogiannis, 2014; Turner, 2014; Francis Stuart et al., 2016). Using cardiac fibroblast-specific Cre lines, it was found that blocking the effects of proinflammatory ILs on cardiac fibroblasts through cardiac fibroblast-specific deletion of IL receptors, *Il1r1* (Bageghni et al., 2019) or *Il17ra* (Chen et al., 2018), both alleviated injury-induced cardiac fibrosis and cardiac function reduction. Interestingly, in both studies, cardiac fibroblast-specific deletion of IL receptor genes reduced the infiltration/activity of immune cells in the injured heart, suggesting that cardiac fibroblasts play an important role in the regulation of post-injury inflammation, which is mediated by IL signaling. In contrast, overexpressing *Il11*, whose expression in fibroblasts is activated by TGFβ, in cardiac fibroblasts caused spontaneous cardiac fibrosis without injury (Schafer et al., 2017).

### Direct Targeting ECM

Being one of the most important characteristics of the fibroblast, the direct regulation of collagen secretion and ECM remodeling in fibroblast is also an important research target. Collagen is first secreted from cells in the form of procollagen. *In vitro* studies identified that the secretion of procollagen required HSP47, a stress-inducible chaperone protein (Nagata et al., 1988; Satoh et al., 1996; Ishida et al., 2006). As expected, the deletion of *Hsp47* in cardiac fibroblasts specifically reduced collagen production but not the general paracrine secretory activity (Khalil et al., 2019). Cardiac fibroblast-specific *Hsp47* deletion significantly reduced cardiac fibrosis and improved heart diastolic function after TAC. However, the reduced collagen accumulation in these mice led to an increase in lethality after MI, owing to the insufficient scar formation (Khalil et al., 2019). This study strongly suggests that the effect of cardiac fibroblast-mediated fibrotic response after cardiac injury can vary significantly among different injury types. While the beneficial effect of fibrotic tissue may outweigh its deleterious effect after an acute injury that causes massive cardiomyocyte death, the effect of interstitial fibrosis in chronic cardiomyopathy may be largely detrimental. Fibronectin is another abundant ECM protein in the heart. Its level in the heart increases significantly after injuries (Heling et al., 2000; Dobaczewski et al., 2006; van Dijk et al., 2008). Cardiac fibroblast-specific *Fn1* deletion led to improved cardiac function, reduced cardiac fibroblast activity, and attenuated cardiac fibrosis and hypertrophy after I/R (Valiente-Alandi et al., 2018), which was likely related to the function of fibronectin in collagen assembly (Sottile et al., 2007). The post-injury ECM remodeling involves collagen crosslinking, which is primarily mediated by isoforms of lysyl oxidases (LOXs). Yang et al. (2016) identified a specific increase in lysyl oxidase-like 2 (LOXL2) level after TAC. Cardiac fibroblast-specific deletion of *Loxl2* significantly improved TAC-induced cardiac function (Yang et al., 2016). Interestingly, besides the expected reduction in fibrosis, the production of TGFβ2 was also reduced in cardiac fibroblasts lacking *Loxl2*, which was found to be mediated by PI3K/AKT signaling (Yang et al., 2016).

### A Re-look at Cardiomyocyte-Expressed Genes

Previous research focusing on cardiomyocytes revealed the cardiomyocyte-specific functions of many genes. The development of cardiac fibroblast-specific gene deletion and overexpression strategies has recently permitted the study of these genes in cardiac fibroblasts. The function of G protein-coupled receptors (GPCRs) in the pathological changes of cardiomyocytes after injuries has been studied extensively (Koch et al., 1995; Raake et al., 2008; Schumacher et al., 2015). However, the role of GPCRs in cardiac fibroblast-mediated fibrosis was only revealed recently. It was reported that cardiac fibroblast-specific deletion of *Grk2* (G protein-coupled receptor kinase 2) reduced the secretion of TNFα by cardiac fibroblasts and cardiac fibrosis after I/R, together with improved cardiac function (Woodall et al., 2016; Travers et al., 2017). Moreover, the function of Hippo pathway, which is known for its inhibitory effect on cardiomyocyte proliferation (Halder and Johnson, 2011; Leach et al., 2017), in cardiac fibroblasts was studied (Xiao et al., 2019). It was found that cardiac fibroblast-specific deletion of Hippo pathway kinases, *Lats1* and *Lats2*, caused spontaneous myofibroblast differentiation (Xiao et al., 2019). This mechanistic study identified that Yap directly activated myofibroblast genes, which was blocked by the phosphorylation of Yap by Lats1/2.

### Novel Transcription Factors

Using cardiac fibroblast-specific Cre lines, novel transcription factors regulating cardiac fibroblast activities have also been identified. Krüppel-like factor 5 (Klf5) is a transcription factor regulating cell differentiation and animal development (Dang et al., 2000; Bieker, 2001; Black et al., 2001). The Nagai group recently reported that the global heterozygous knockout of *Klf5* reduced cardiac hypertrophy and fibrosis induced by Ang II infusion (Shindo et al., 2002). A later study conducted by the same group found that cardiac fibroblast-specific *Klf5* deletion ameliorated cardiac hypertrophy induced by moderate-intensity pressure overload, which, however, was not observed in cardiomyocyte-specific *Klf5* knockout mice (Takeda et al., 2010). It was found that KLF5 activated IGF-1 secretion from cardiac fibroblasts, which then served as the mediator in the crosstalk between cardiac fibroblasts and cardiomyocytes (Takeda et al., 2010). Another recent study combining TOMO-seq and transcription factor binding site analysis identified SOX9 as a potential novel transcription factor activating the expression of fibrotic genes in the heart (Lacraz et al., 2017), which might explain the amelioration in MI-induced cardiac dysfunction, fibrosis, and dilation in mice with cardiac fibroblast-specific deletion of *Sox9* (Scharf et al., 2019).

### Epigenetic Regulation

The study of epigenetic regulation of myofibroblast differentiation and cardiac fibrosis has also benefited from cardiac fibroblast-specific Cre lines. It was recently found that a metabolic switch to glycolysis during myofibroblast differentiation due to the shutdown of mitochondrial calcium uniporter complex increased the production of α-ketoglutarate which then enabled histone demethylation at myofibroblast gene loci, promoting myofibroblast differentiation (Lombardi et al., 2019). Deletion of mitochondrial calcium uniporter (*Mcu*) in fibroblasts, blocked mitochondria calcium influx, and therefore promoted the metabolic switch. As a result, cardiac fibroblast-specific deletion of *Mcu* attenuated myofibroblast differentiation and improved cardiac function after cardiac injury (Lombardi et al., 2019). Moreover, another study found that microRNA, miR-33 regulated cholesterol metabolic gene expression in cardiac fibroblasts, which was important for cardiac fibroblast proliferation. Cardiac fibroblasts lacking *miR-33* had reduced proliferation due to altered lipid raft cholesterol content. *Postn-Cre*-induced deletion of *miR-33* in mice led to decreased cardiac fibrosis (Nishiga et al., 2017).

## The Clinical Implication of Cardiac Fibroblast Lineage-Tracing

Studies have shown that simply depleting cardiac fibroblasts from the heart after an injury usually causes detrimental effects (Kanisicak et al., 2016; Fu et al., 2018). More and more evidence generated using cardiac fibroblast lineage-tracing tools and cardiac fibroblast-specific Cre lines have suggested an increasing number of cardiac fibroblast-expressed genes as potential targets for treating cardiac diseases. Thus, manipulation of the cardiac fibroblast gene expression profile may be a feasible approach. Systemic delivery of traditional medication that regulates the expression of certain genes or the activity of relevant signaling pathways has been tested in clinical trials for cardiac fibrosis treatment, which, however, usually indiscriminately targets all the cells in the body and has unintended targets, often leading to severe side effects. For instance, animal studies have shown the anti-cardiac fibrosis effects of TGFβ inhibitors such as pirfenidone and tranilast (Edgley et al., 2012). However, a hepatic adverse effect was identified in a clinical trial testing the therapeutic effect of tranilast in restenosis caused by percutaneous coronary intervention (Holmes et al., 2002). The development of gene therapies, especially the adeno-associated virus (AAV)-mediated gene therapy (Finer and Glorioso, 2017), has now made it possible to specifically manipulate gene expression in certain cell types. Dependent on the transgene selected, gene therapy allows both direct overexpression and knockdown of a certain gene in the cell (Eaton et al., 2002; Chadderton et al., 2009; Samulski and Muzyczka, 2014). Over the years, different AAV serotypes that specifically target different organs have been reported (Wu et al., 2006). A greater specificity has been achieved using cell type-specific promoters to drive the expression of transgenes (Peel et al., 1997; Dashkoff et al., 2016; Hanlon et al., 2017). Using this approach, multiple groups have reported successful specific expression of transgenes in cardiomyocytes (Aikawa et al., 2002; Pleger et al., 2007; Pacak et al., 2008). Recently, AAV carrying a *Postn* promoter-driven transgene has also been used to specifically induce transgene expression in myofibroblasts in the heart (Piras et al., 2016), strongly suggesting a bright future of this treatment strategy.

## Conclusion

The great plasticity of cardiac fibroblasts allows them to quickly respond to the injury/disease signal and then contribute to the alteration and remodeling of the myocardial environment. Being the most abundant cell type in the heart, it is undoubted that the cardiac fibroblast is an excellent target for treating cardiac diseases. The development of cardiac fibroblast lineage-tracing mouse lines has led to a huge acceleration in cardiac fibroblast research. Using these tools, researchers have been able to reveal the developmental origin of cardiac fibroblasts and their differentiation pathways in injuries and diseases. Even though an increasing number of signaling pathways have been suggested to play a role in the regulation of cardiac fibroblast activities and differentiation, more studies are still required to reveal the details of these regulations, such as how these signaling pathways specifically regulate the beneficial and deleterious effects of cardiac fibroblasts and how they crosstalk with each other. Moreover, due to difference in the effect of fibrosis on cardiac tissue healing and function after different cardiac injuries indicated in recent studies (Khalil et al., 2019), more work comparing the effects of the cardiac fibroblast-specific knockout and overexpression of the same genes in different disease/injury models are also needed. These studies are particularly important for the selection of appropriate treatment target. In addition, the current mechanistic understanding of the plasticity of cardiac fibroblasts is largely limited to myofibroblast differentiation. Signaling pathways regulating other differentiation potentials of cardiac fibroblasts may also deserve more attention because of the increased evidences of these differentiations in diseased hearts. In addition, even though many of the cardiac fibroblast lineage-tracing tools have shown high fibroblast-specificity in the heart, most of the promoters/loci used in these cardiac fibroblast lineage-tracing tools are also active in fibroblasts and similar cell types residing in other organs (Quaggin et al., 1999; Kanisicak et al., 2016). Thus, further studies are required to engineer those promoters/loci to achieve absolute cardiac fibroblast-specificity, which is required for the specific regulation of target genes in cardiac fibroblasts in gene therapy. However, considering the relatively short history of the modern lineage-tracing system, it is expected that the progress of this research area will further accelerate. In summary, the advances in cardiac fibroblast lineage-tracing studies and their potential application in gene therapy may shed light on novel treatment approaches for cardiac diseases.

## Author Contributions

XF was the primary author who is responsible for the design and writing of the manuscript. QL, CL, YL, and LW assisted with the writing of the manuscript.

## Conflict of Interest

The authors declare that the research was conducted in the absence of any commercial or financial relationships that could be construed as a potential conflict of interest.
